# Therapeutic Optimization of *Pseudomonas aeruginosa* Phages: From Isolation to Directed Evolution

**DOI:** 10.3390/v17070938

**Published:** 2025-06-30

**Authors:** Sara Bolognini, Caterina Ferretti, Claudia Campobasso, Elisabetta Trovato, Magda Marchetti, Laura Rindi, Arianna Tavanti, Mariagrazia Di Luca

**Affiliations:** 1 Department of Biology, University of Pisa, 56126 Pisa, Italy; s.bolognini1@student.unisi.it (S.B.); claudia.campobasso@phd.unipi.it (C.C.); e.trovato3@studenti.unipi.it (E.T.); arianna.tavanti@unipi.it (A.T.); 2 Department of Medical Biotechnologies, University of Siena, 53100 Siena, Italy; 3 Department of Translational Research and New Technologies in Medicine and Surgery, University of Pisa, 56126 Pisa, Italy; c.ferretti15@studenti.unipi.it (C.F.); laura.rindi@unipi.it (L.R.); 4 National Centre for Innovative Technologies in Public Health, National Institute of Health, 00161 Rome, Italy; magda.marchetti@iss.it

**Keywords:** direct phage evolution, phage–antibiotic combination, phage therapy, lung infection, cystic fibrosis

## Abstract

*Pseudomonas aeruginosa* is a major opportunistic pathogen with high levels of antibiotic resistance. Phage therapy represents a promising alternative for the treatment of difficult infections both alone and in combination with antibiotics. Here, we isolated and characterized three novel lytic myoviruses, Cisa, Nello, and Moonstruck. Genomic analysis revealed that Cisa and Nello belong to the *Pbunavirus* genus, while Moonstruck is a novel *Pakpunavirus* species. All lacked lysogeny, virulence, or resistance-associated genes, supporting their therapeutic suitability. Phage Nello and Moonstruck were active against *P. aeruginosa* Pa3GrPv, isolated from a patient with lung infection candidate for phage therapy. Moonstruck exhibited superior lytic activity with ciprofloxacin sub-MIC value (0.125 µg/mL), achieving bacterial suppression for 48 h. However, to improve the lytic efficacy of the phages on the clinical isolate, phage adaptation via serial passage was investigated. The killing efficacy of Nello was enhanced, whereas Moonstruck showed a less consistent improvement, suggesting phage-specific differences in evolutionary dynamics. Sequencing of the evolved phages revealed point mutations in tail-associated genes, potentially linked to a better phage–host interaction. These results support the use of phage–antibiotic combinations and directed evolution as strategies to enhance phage efficacy against drug-resistant infections. Overall, these findings support the therapeutic potential of the newly isolated phages in treating *P. aeruginosa* lung infections.

## 1. Introduction

Antimicrobial resistance (AMR) is an urgent global public health threat caused by the misuse and over-use of antibiotics in different fields, such as animal husbandry, the food industry, and the clinical field [1]. Antibiotics pressure adds to the selection of drug-resistant bacterial clones and difficult-to-eradicate infections [1].

An increasing treatment challenge for healthcare institutions and public health worldwide is represented by a group of pathogens, *Enterococcus faecium*, *Staphylococcus aureus*, *Klebsiella pneumoniae*, *Acinetobacter baumannii*, *Pseudomonas aeruginosa*, *Enterobacter species*, and *Escherichia coli*, collectively referred to by the acronym ESKAPEE. They cause the most difficult-to-treat hospital-acquired infections worldwide [2].

In particular, *P. aeruginosa* is a ubiquitous opportunistic Gram-negative pathogen characterized by rod-shape and motility [3]. *P. aeruginosa* can cause acute or chronic respiratory infections, especially in immunocompromised individuals and patients with cystic fibrosis, resulting in increased morbidity and mortality [3]. As a soil bug *Pseudomonas aeruginosa* displays intrinsic resistance to some antibiotic classes [4]. Moreover, the genetic plasticity and adaptability of *P. aeruginosa* facilitate the selection of antibiotic-resistant bacterial clones, for instance, through mutations induced by drug selective pressure in chromosomal genes involved with the mechanism of action of an antibiotic or through the acquisition of external genetic determinants of resistance via horizontal gene transfer [5].

The threat of AMR has revitalized research in alternative solutions to antibiotics and phage therapy is re-emerging as a promising solution [1]. Bacteriophages are viruses that specifically infect bacteria and are the most abundant organisms on the planet, with an estimated 10^31^ bacteriophage particles in the biosphere [1]. Indeed, environmental phages can be isolated from samples of water, soils, animals, plants, human body and consequently in waste downstream from human or animal communities and sewage treatment plants [1,6]. In choosing phages for therapeutic use there are several characteristics that should be considered. First, the phages should kill the specific bacteria efficiently in vitro without significant levels of survivors; furthermore, the phage should be easy to propagate and to purify at high-titer preparations. It is recommended to select phages that are stable at storage conditions, to reduce the risk of the loss of infectivity; furthermore, the preparation must be sterile and not contain harmful contaminants such as endotoxins. Lastly, phage genomes should not contain genes encoding for toxins or associated with antibiotic resistance and must not be capable of acting as transducing phages [7].

In the environment, phages and bacteria co-exist and co-evolve in such a way that none of the actors take over the other. In fact, one of the common observations in the application of phage therapy is the emergence of bacterial resistance to phages. However, on the other hand, phages also adapt to the evolving bacteria and overcome or minimize microbial resistance onset [8].

Phages are biological entities that can evolve by reciprocally adapting to changes in their hosts (co-evolution) to maintain the ability to infect them. As phages adjust naturally in response to bacterial resistance, there is also the possibility to harness this inherent evolutionary potential by pre-emptively co-evolving phages with their target. This novel “phage training” approach suggests that, by experiencing the evolution of resistance in their host, phages can be selectively adapted to become more effective at overcoming host defenses. These trained phages “from the future” can be used against the native, non-co-evolved bacteria “from their past” which should then be susceptible to infection by these phages [9,10].

The main aim of this study was to isolate and characterize new bacteriophages targeting *P. aeruginosa* strains, to include in our phage collection. Then, two of them were selected to be trained against a bacterial isolate causing lung infection in a patient candidate for phage therapy. Finally, we investigated the mutations that emerged in the evolved phage variants to highlight the main genes under selective pressure that play a key role in the phage–bacteria interaction.

## 2. Materials and Methods

### 2.1. Pseudomonas aeruginosa Strains

Two *P. aeruginosa* strains, Pa3host and Pa3GrPv, were included in the study. Pa3host come from the collection of clinical isolates of the microbiology laboratory of the University of Pisa. It was selected for phage isolation from the environment and used as a phage host strain, while Pa3GrPv was isolated from a patient with pulmonary infection, a candidate for phage therapy.

*P. aeruginosa* strains were cultured in Luria–Bertani (LB) broth (Sigma-Aldrich, St. Louis, MO, USA) at 37 °C with shaking. Overnight-grown bacteria were stocked at −80 °C. When plated, bacteria were diluted in sterile phosphate-buffered saline (PBS: 137 mM NaCl, 2.7 mM KCl, 10 mM Na_2_HPO_4_, 1.8 mM KH_2_PO_4_) and 20 μL was plated on LB 1.5% agar (Sigma-Aldrich, St. Louis, MO, USA) plates.

### 2.2. Isolation of New Pseudomonas aeruginosa Phages

Phages were isolated from environmental water samples collected in Pisa and Lucca, Tuscany, following an already-described enrichment procedure [11]. For phage enrichment, liquid samples were centrifuged at 4000× *g* for 20 min, supernatants were filtered (0.22 micron), and 50 mL of the filtrate was incubated under weak agitation (80 rpm) for 24 h at 37 °C in 250 mL Erlenmeyer flasks with 50 mL of 2-fold-concentrated LB broth (added with 0.1 mM of CaCl2) and 50–60 μL of overnight-grown bacterial suspension.

The enrichment solutions were centrifuged (4500× *g* for 30 min), filtered with a syringe filter (0.22 μm pore diameter), and spotted on a bacterial lawn to identify phage lysis. Following an overnight incubation at 37 °C, different sizes and morphologies of isolated plaques were visible on the bacterial lawn. Single plaques were isolated by picking with a sterile tip, streaking it on a new LB agar plate with bacterial lawn, making a puncture line, then streaking downwards with a sterile paper strip partially overlapping the previous streak to dilute the phages and isolate plaques. Plates were incubated overnight at 37 °C. This procedure was repeated until the plaques that resulted were uniform. Subsequently, phages were eluted overnight adding 5 mL of saline magnesium buffer (SM: 50 mM Tris-HCl, 8 mM magnesium sulfate, 100 mM sodium chloride- pH 7.5), and rocking. Then, SM buffer with eluted phages was collected, centrifugated at 5000× *g* for 15 min, and filtered (0.22 μm). Then, the phage titer was determined using a double-layer spot assay [12]. Briefly, 10 μL of phage 10-fold serial dilutions was spotted onto the bacterial lawn and incubated overnight at 37 °C. Visible isolated plaques were counted to determine plaque-forming units (PFU/mL).

### 2.3. Transmission Electron Microscopy (TEM) Imaging

Aliquots of 10 μL of each phage sample were adsorbed onto formvar/carbon-coated 400-mesh copper grids (Agar Scientific, Essex, UK). Excess liquid was removed using filter paper. Subsequently, 10 μL of 2% (*w*/*v*) phosphotungstic acid (pH 7.0) was applied to the grids for 30 s for negative staining. The samples were examined using an FEI/Philips EM 208S transmission electron microscope (FEI, Eindhoven, The Netherlands) operating at an accelerating voltage of 100 kV and equipped with an acquisition system/Megaview SIS camera (Olympus, Hamburg, Germany).

### 2.4. Bacteriophage Genome Extraction and Analysis

The phage genome was extracted as previously described [13]. Briefly, 180 μL of phage lysate was supplemented with 20 μL of 10X DNaseI (Thermo Fisher Scientific, Waltham, MA, USA) buffer and 10 μL of DNaseI (1 U/μL) (Thermo Fisher Scientific, Waltham, MA, USA), RNA-free, was added and incubated at 37 °C for 30 min. Subsequently, 20 μL of 50 mM EDTA and 20 μL of 1% SDS were added. Then, 10 μL of proteinase K (>600 U/mL) (Thermo Fisher Scientific, Waltham, USA) was added with incubation at 55 °C for 45 min. To purify the phage DNA, a Zymo Research DNA Clean & Concentrator kit was used according to the manufacturer’s instructions. A NanoDrop Lite (ThermoFisher Scientific, Waltham, USA) was used to quantify the extracted genome and the DNA samples were stored at −20 °C.

Illumina sequencing libraries were set up using the Nextera Flex DNA library kit. The obtained raw sequences were uploaded to the BV-BRC platform (https://www.bv-brc.org; accessed on 27 June 2025). Genome assembly was performed with the aid of the Unicycler tool (v0.4.8), which is available on the website, and the output was visually inspected, using the Bandage software (v 0.8.1), to confirm the presence of a complete contig [14]. The resulting FASTA files were then analysed on BLASTn (https://blast.ncbi.nlm.nih.gov/Blast.cgi; accessed on 27 June 2025) to identify the most similar phage-genome already in the NCBI database.

Using the EASYFIG (v2.1) tool the FASTA sequence was aligned with the genome of the best hit to determine the starting points [15]. When needed, the start of the phage genome FASTA files was manually corrected with UGENE (v49.1) [16]. The updated FASTA files were reuploaded on the BV-BRC platform, where putative coding sequences (CDSs) were identified using RASTtk (v1.3.0) [17]. This initial annotation was further refined manually, running BLASTp on each predicted protein, and a function was appointed based on homology with proteins in the NCBI database.

The packaging strategy was assessed comparing the protein sequence of the terminase large subunits of these three new phages to 43 terminase large subunits from other phages whose packaging strategies were already experimentally verified [18].

Moreover, the possible presence of antibiotic-resistance or virulence genes was also evaluated with ABRicate on the Galaxy platform (https://usegalaxy.org.au/; accessed on 27 June 2025) using the ResFinder 4.1 database [19], VirulenceFinder 2.0 [20,21], CARD [22], and the NCBI Antimicrobial Resistance Gene Finder 4.0 [23].

The phages’ genome information is deposited in GenBank—Cisa and Nello with accession numbers PV464114 and PV464115, and Moonstruck under PV464116.

### 2.5. Phenotypical Characterization of Bacteriophages

One-step growth curves were defined according to Pires et al., with some modifications [24]. Briefly, 10 mL of exponential phase culture was centrifugated (5000× *g*, 4 °C, 20 min), the supernatant discarded, and the pellet resuspended in 5 mL of fresh LB broth. Subsequently, 5 mL of phage lysate 10^6^ PFU/mL (MOI = 0.01) was added to the bacterial culture and incubated for 5 min at 37 °C with shaking. After incubation, the culture was centrifugated at 5000× *g* for 20 min and the supernatant, containing not-adsorbed phages, was discarded. Subsequently, the pellet was resuspended in 10 mL of fresh LB broth and 200 μL was taken before incubation at 37 °C and shaking to represent the phage titer at time-point zero. Subsequently, a sample was taken at different time-points for each phage: 0, 5, 10, 15, 20, 30, 40, 50, 60, 70 for phage Nello and 0, 5, 10, 15, 20, 30, 40, 50, 60, 70, 80, 90 for Moonstruck. For each sample, the PFU/mL was evaluated.

The pH stability was assessed according to the Fujiki et al. protocol with some modifications [25]. The pH of neutral SM buffer was adjusted using HCl or NaOH. Solutions of SM buffer at different pH, ranging from 3 to 12, were prepared, and for each, the phage suspension (10^8^ PFU/mL) was incubated for 1 h at 37 °C in a microcentrifuge tube and then diluted and spotted on the bacterial lawn.

### 2.6. In Vitro Activity of Bacteriophages and Ciprofloxacin Against Planktonic P. aeruginosa

An antimicrobial susceptibility test of ciprofloxacin was performed by broth microdilution according to EUCAST guidelines [26]. Experiments were performed in a 96-well round bottom plate containing 100 µL of exponential phase bacteria (1 × 10^6^ CFU/mL) in cation-adjusted Mueller–Hinton broth (CAMHB) (Sigma-Aldrich, St. Louis, USA) and two-fold serial dilutions of ciprofloxacin. A growth control (bacteria without treatment) was also added. The plate was incubated statically overnight at 37 °C. For the growth kinetic assay, a plate containing exponential-phase bacteria treated with phages Nello and Moonstruck alone and in combination (MOI 10) with ciprofloxacin (0.125 and 0.5 µg/mL) was read with a TECAN Sunrise microtiter plate reader (Tecan, Männedorf, Switzerland) equipped with a 600 nm filter.

### 2.7. Direct Evolution Protocol and Phages Bactericidal Killing Assay

To improve the lytic activity of Nello and Moonstruck phages versus the Pa3GrPv strain, fifteen rounds of evolution, each of five days of incubation, were carried out. This was performed by serial rounds of 5-days incubation since reported by Tkhilaishvili et al. [27].

**Figure 1:**
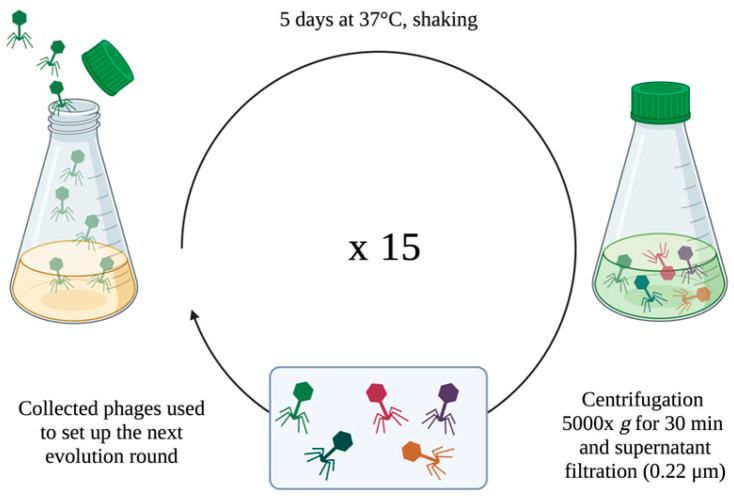
Schematic representation of the workflow for the direct evolution method used to evolve phage Nello and Moonstruck on Pa3GrPv. Created with Biorender (https://www.biorender.com; accessed on 27 June 2025).

A schematic illustration of the protocol is represented in Figure 1.

Exponential growing bacteria (final concentration of 10^6^ CFU/mL) were combined with the phage suspension (final titer of 10^6^ PFU/mL) at an MOI of 1, in a final volume of 20 mL of LB broth in a Corning^®^ 125 mL Erlenmeyer Flask. Flasks were incubated at 37 °C in agitation for 5 days. Subsequently, the suspension was centrifuged at 5000× *g* for 30 min and the supernatant filtered with 0.22 μm filter to collect phages, then stored at 4 °C. Collected phages were titered and used to set up the following 5-day evolution round against the wild-type clinical isolate; 15 rounds were conducted. After each round, the titer of the trained phages was evaluated by double-layer assay to assess their replication during the incubation with bacteria.

The evolved phages were tested against Pa3GrPv to compare their lytic ability to that of the original phages. A bacterial culture in exponential growth phase and bacteriophage lysate (MOI 1) was incubated under shaking for 24 h at 37 °C overnight. Then, the bacterial load (CFU/mL) was evaluated by colony counting.

### 2.8. Statistical Analysis

All graphs and statistical analysis were performed on Prism software (version 8.0.2; GraphPad Software, La Jolla, San Diego, CA, USA). For the heat stability assay and the pH stability assay, an unpaired Student’s *t*-test was performed. For lysis kinetics and lytic activity assay one-way ANOVA multiple comparisons using Tukey correction was performed. For all tests, differences were considered significant when *p*-values were <0.05.

## 3. Results

### 3.1. Imaging and Genomic Features of Newly Isolated Pseudomonas aeruginosa Phages

Three bacteriophages, named Cisa, Nello, and Moonstruck, were isolated from environmental samples, especially stagnant and river water from two different cities in Tuscany, Pisa, and Lucca. In particular, Cisa was isolated from a pond near the Lucca wastewater treatment plant, Nello from the Arno River, and Moonstruck from pond water near The Santa Chiara hospital in Pisa.

**Figure 2:**
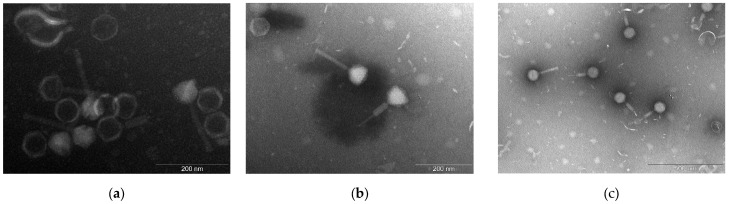
Transmission electron microscopy images of isolated phages: (**a**) Cisa, (**b**) Nello, and (**c**) Moonstruck, negatively stained by 2% phosphotungstic acid.

In Figure 2, Transmission Electron Microscopy (TEM) imaging showed they were all myoviruses characterized by a long contractile tail and an icosahedral head [28]. In the same picture the contracted and not contracted tail was observed for each phage.

The genomes of the three phages were determined by Illumina sequencing (Table 1). All phages have a dsDNA genome, specifically, Cisa and Nello of around 65 kb, and Moonstruck of 92 kb. Phage Cisa and Nello both showed a 97.18% identity with *P. aeruginosa* phage misfit (MT119367.1) with a 100% query coverage. Regarding phage Moonstruck, the most similar sequence was *P. aeruginosa* phage PAK_P4 (NC_022986.1) with a query cover and identity of 97% and 96.42%, respectively. For all three phage assembled genomes, their respective BLAST best hit was used as the reference sequence for their orientation and annotation.

According to Turner et al., newly isolated bacteriophages can be classified as new species when the similarity to the closest phage genome sequence already present in literature is below 95% [29]. In the case of phage Cisa and phage Nello, they showed a similarity of 97.18% to their best hit, classifying them under the species of phage misfit, belonging to the *Pbunavirus* genus. Conversely, phage Moonstruck showed a similarity of 93.52% to PAK_P4 and it can be considered as a new phage species belonging to the *Pakpunavirus* genus. The three bacteriophages were annotated to identify the ORFs and characterize the encoded proteins (Table 2). Phages Cisa and Nello carried a total of 107 coding sequences (CDSs) and for almost 50% of them a putative function was predicted. No tRNA sequences were found in their genomes.

The genome of phage Moonstruck carried 199 CDSs, 77 of which encoded for proteins with a predicted function, and 12 tRNAs.

By aligning the terminase sequence of Cisa, Nello and Moonstruck to 43 other terminases with known packaging methods, the highest similarity observed was with the terminase of *Enterobacteria* phage CUS-3 and *Shigella* virus Sf6 (with coverage/identity values of 97%/35.81% and 94%/36.43% for Cisa and Nello, and 96%/35.81% and 96%/35.81% for Moonstruck), two viruses which use a headful packaging strategy. Therefore, the newly isolated bacteriophages probably all use the same packaging strategy. No genes associated with a lysogenic lifestyle, such as integrase or repressors, were identified in the three phage genomes, therefore indicating their strictly virulent nature. Moreover, no antimicrobial resistance genes were detected in any of the phage genomes. Similarly, no known virulence factors were identified, suggesting that these phages are unlikely to contribute to horizontal gene transfer of harmful traits.

**Figure 3:**
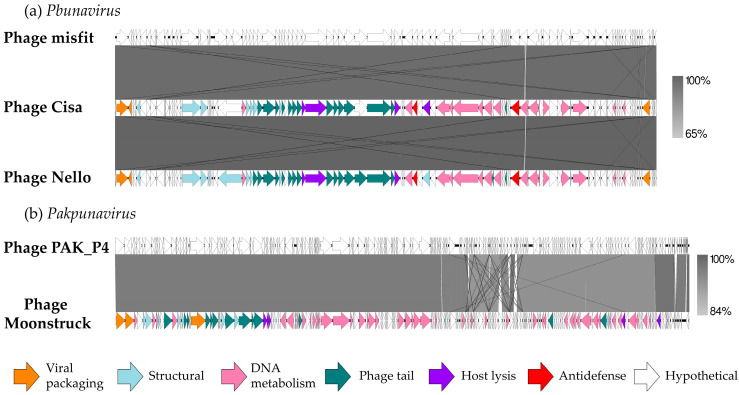
Genome representation of isolated phages and comparison with their best-hit sequence (**a**) Phage Cisa and Phage Nello compared to *Pbunavirus* phage misfit; (**b**) Phage Moonstruck compared to *Pakpunavirus* phage PAK_P4. The different modules are DNA metabolism (pink); packaging (orange); host lysis (purple); structural protein (light blue); tail fiber proteins (teal); antidefense protein (red); hypothetical protein and unknown function (white); and tRNA (light green). Arrows represent open reading frames with direction. Level of acid nucleic identity (%) is shown by the gradient scales.

In Figure 3, a comparison of the DNA of the three new bacteriophages with their respective best hit is shown to highlight synteny between phages belonging to the same genus.

The predicted proteins identified were categorized into five functional classes: DNA metabolism and replication (including DNA repair and modification), virion structure (such as head and tail morphogenesis), viral packaging, host lysis, and hypothetical proteins. The genes encoding for structural proteins were clustered together, as well as the nucleotide metabolism genes.

Figure 3a clearly displays a high level of similarity (99.94%) between the *Pbunavirus* phages, either between the new ones and the reference phage but also among them. They showed a similar organization of functional clusters linked to phage morphogenesis, lysis, and DNA processing. Between the phage Cisa and Nello sequences, 17 missense mutations were identified, some of them carried by the tail fiber and the baseplate protein-associated genes (Table S1, Supplementary Materials).

Phage Moonstruck was also compared to its reference (Figure 3b) and most of the differences between the two genomes were found in the DNA metabolism functional cluster, while the rest of the genome showed conserved gene sequences.

The genome sequences of these three phages are available under the accession numbers PV464114, PV464115, and PV464116 in the NCBI database.

### 3.2. Phenotypical Characterization of the Phages

Since the genomic comparison between the two *Pbunavirus* phages showed a very high similarity, we considered them as two variants of the same bacteriophage., Therefore, we arbitrary selected phage Nello for the following phenotypical characterization and further experiments.

**Figure 4:**
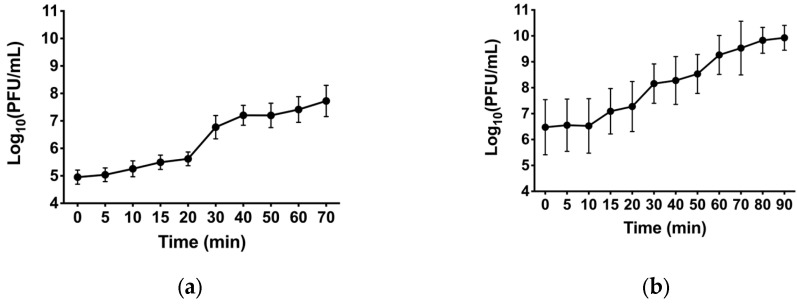
One-step growth curve of bacteriophages (**a**) Nello and (**b**) Moonstruck on *P. aeruginosa* Pa3host at 37 °C in LB broth with an MOI of 0.01. In the graphs, the Log_10_ (PFU/mL) are shown at different time-points after infection.

First, a one-step growth curve was performed on their bacterial host, Pa3host, to study the latent period (the average time between phage adsorption and the start of lysis) and the burst size (the number of viral particles released per cell during infection) (Figure 4).

Phage Nello showed a latent period of around 20 min where the phage titer was constant around 10^5^ PFU/mL. The plateau was reached in 40 min and the burst varied from around 10^5^ to 10^7^ PFU/mL (Figure 4a). Phage Moonstruck exhibited a latent period of 10 min where the phage titer was stable around 10^6^ PFU/mL, showing the plateau after 70 min, with the burst reaching 10^10^ PFU/mL (Figure 4b).

**Figure 5:**
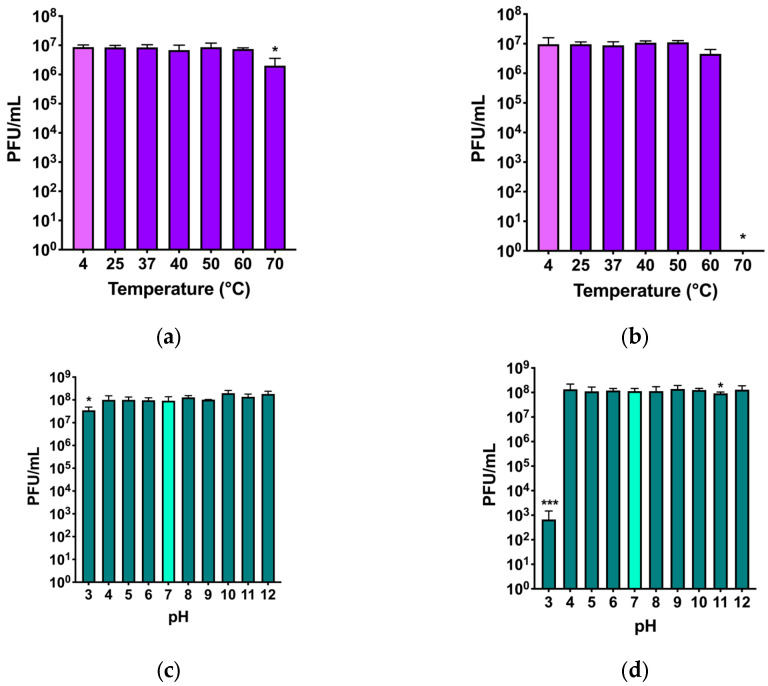
Phage titer of (**a**) Nello and (**b**) Moonstruck evaluated after exposure to different temperatures (4 °C, 25 °C, 37 °C, 40 °C, 50 °C, 60 °C, 70 °C) for 1 h. A Student’s *t*-test was performed between the phage titer at 4 °C, stocking temperature, and the phage titer after 1 h, for each temperature. * *p* ≤ 0.05. N = 3. PFU/mL after 1 h incubation of (**c**) Nello and (**d**) Moonstruck in SM buffer with different pH (3, 4, 5, 6, 7, 8, 9, 10, 11, 12). A Student’s *t*-test was performed between the phage titer at pH 7, considered the starting titer for the evaluation of eventual decreases, and the phage titer after 1 h for each pH value. * *p* ≤ 0.05; *** *p* ≤ 0.001.

The stability of the two phages under physical or chemical stresses was evaluated testing the maintenance or reduction in the initial phage titer after exposure of phages to high temperatures and acidic and basic pH, respectively (Figure 5).

For phage Nello, a stable titer at all temperatures was observed, although at 70 °C, a slight decrease of almost 1log_10_ was observed (Figure 5a). For phage Moonstruck, the titer was stable at all temperatures up to 60 °C, while at 70 °C the phage titer dropped, and no plaques could be counted (Figure 5b).

The pH stability of phage Nello was not affected by incubation at the tested pH values, except for the incubation at pH 3, which showed a decrease of almost 1log_10_ compared to the initial titer (Figure 5c). For phage Moonstruck, a decrease of 5log_10_ of the initial titer was observed at pH of 3, while the phage titer was stable at all other tested pH values (Figure 5d).

### 3.3. Growth Kinetics of Bacteria Treated with Phages and Ciprofloxacin, Alone and in Combination

**Figure 6:**
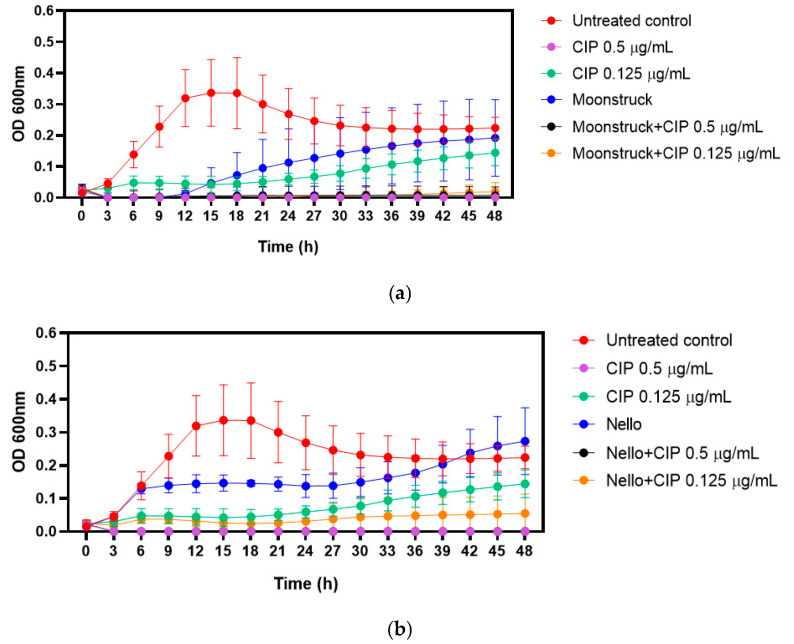
Growth kinetics of Pa3GrPv treated with Moonstruck (**a**), Nello (**b**), and ciprofloxacin (CIP), alone and in combination. OD_600_ values are reported on the y-axis while time in hours is reported on the x-axis.

Based on clinical considerations, growth kinetics were performed to evaluate the effect of phages–ciprofloxacin combination against Pa3GrPv. Phages Moonstruck and Nello were tested at MOI 10, while ciprofloxacin was applied at its MIC (0.5 µg/mL) and two-fold serial dilutions (0.125 µg/mL) below the MIC (Figure 6).

The MIC of ciprofloxacin, determined by visual inspection following broth microdilution, was confirmed by measuring the optical density overtime. As expected, ciprofloxacin at 0.5 µg/mL (MIC value) suppressed bacterial growth for up to 48 h.

When tested alone, phage Moonstruck inhibited bacterial growth for up to 12 h, after which regrowth was observed (Figure 6a). In contrast, phage Nello showed lower bactericidal activity (Figure 6b).

The combination of each phage with sub-MIC concentration (0.125 µg/mL) ciprofloxacin determined a lower OD value in comparison to the growth control and bacteria treated with either phages or ciprofloxacin (at 0.125 µg/mL) alone, suggesting that both phages and antibiotic enhanced their bactericidal activity. Notably, Moonstruck in combination with ciprofloxacin was able to sustain bacterial suppression for up to 48 h. In addition, both phages tested with ciprofloxacin at MIC did not determine bacterial growth, suggesting no antagonistic effect between phages and antibiotic.

### 3.4. Phage Replication During Evolution and Assessment of Their Lytic Potential

**Figure 7:**
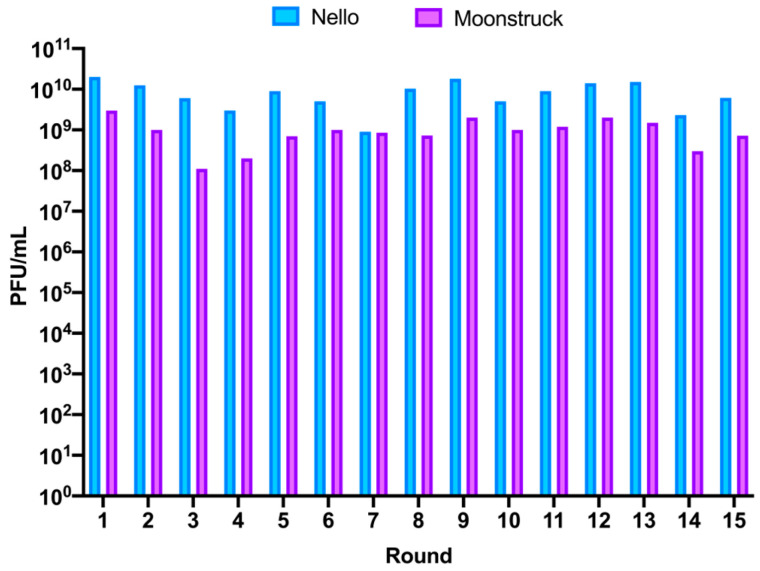
Titer of phages Nello and Moonstruck collected from 15 rounds of evolution (x-axis) expressed in PFU/mL (y-axis). Blue columns show the phage Nello titers while in violet are shown the Moonstruck titers.

Phages Nello and Moonstruck were independently trained using a phage evolution protocol that involved repeated rounds of infection of evolved phages on clinical isolate Pa3GrPv. This was achieved by co-incubating the phages with the host for 5 days for each of the fifteen rounds. On the fifth day of incubation, phages were collected and titered to evaluate the amount of phage particles produced in the round after incubation (Figure 7). For phage Nello, the titer of the collected phages from rounds 1, 2, and 5 (R1, R2 and R5) was of 10^10^ PFU/mL, alternated by rounds in which a slight drop in phage titer was observed of about 0.5log_10_ (R3, R4 and R6) and 1log_10_ for round 7 (R7). For the following rounds, an increase to 10^10^ PFU/mL was observed, while the last two rounds, 14 and 15 (R14 and R15), showed a slight decrease to around 10^9^ PFU/mL. Therefore, for phage Nello, the production of phage particles at each round varied between 10^9^ and 10^10^ PFU/mL, corresponding to an increase of 3–4 log_10_ compared to the starting phage titer of 10^6^ PFU/mL used to infect bacteria at each round.

For phage Moonstruck, firstly, the titer of the collected phages for rounds 1 and 2 (R1 and R2) was 10^9^ PFU/mL, then a decrease of 1log10 was observed for rounds 3 and 4 (R3 and R4). Then, the titers of the following rounds increased to around 10^9^ PFU/mL, except for the last two rounds, 14 and 15, when the phage titer decreased to 3 × 10^8^ and 7 × 10^8^ PFU/mL, respectively. Overall, for Moonstruck, the phage progeny produced at each round varied from 10^8^ to 10^9^ PFU/mL, representing an increase of 2–3 log_10_ in respect of the starting phage titer of 10^6^ PFU/mL added at each round.

**Figure 8:**
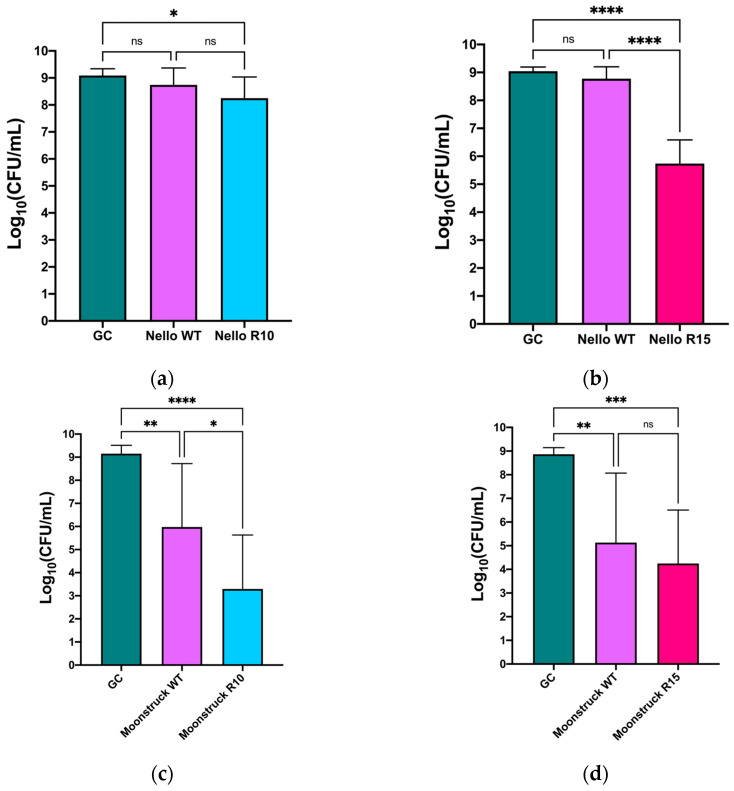
Bacterial load, expressed as CFU/mL, counted for each different condition after the 24 h treatment of *P. aeruginosa* Pa3GrPv with phages Nello and its evolved mutants (**a**) of round 10 and (**b**) of round 15, and Moonstruck and its evolved mutants (**c**) of round 10 and (**d**) of round 15. The one-way ANOVA test with Tukey multiple comparisons was performed for all conditions with the other. * *p* ≤ 0.05; ** *p* ≤ 0.01; *** *p* ≤ 0.001; **** *p* ≤ 0.0001; ns *p* > 0.05.

The bactericidal activity of evolved phages was assessed by comparing their effects to both a growth control (no phage) and the wild-type phage, to determine any significant variations in efficacy. Figure 8 shows the CFU/mL values of *P. aeruginosa* Pa3GrPv incubated with either phage Nello or phage Moonstruck and their mutants from round 10 (Figure 8a,c) and round 15 (Figure 8b,d). Treatment with Nello R10 resulted in around a 1log_10_ reduction in bacterial load compared to the growth control (*p* ≤ 0.05) and in respect to the wild-type phage treatment, though here the reduction was not statistically significant. In contrast, phage Nello R15 induced a reduction of approximately 3log_10_ CFU/mL with respect to the growth control (*p* ≤ 0.0001) (Figure 8b). Furthermore, a difference of about 3log_10_ in bacterial load could be observed between Nello R15 and the wild-type phage (*p* ≤ 0.0001).

Phage Moonstruck, both wild-type and its evolved variants (R10 and R15), exhibited trends similar to those observed for phage Nello and its mutants (Figure 8c,d). Specifically, Moonstruck R10 caused a statistically significant reduction in bacterial load of approximately 7log_10_ CFU/mL compared to the growth control (*p* ≤ 0.0001) (Figure 8c). A reduction of 3log_10_ in the bacterial count was also found between treatment with Moonstruck R10 and the wild-type phage (*p* ≤ 0.05). Moonstruck R15 reduced the bacterial load to approximately 10^4^ CFU/mL, corresponding to a reduction of approximately 5log_10_ CFU/mL relative to the growth control (*p* ≤ 0.001) (Figure 8d). The difference in CFU/mL between treatments with the wild-type and R15 phages was of around 1log_10_ but this result was not statistically significant.

### 3.5. Growth Kinetics of Round 10 and 15 of Phage Nello and Moonstruck

The growth kinetics of wild-type phages Nello and Moonstruck and their mutants, along with their evolved derivatives from rounds 10 and 15 (R10 and R15) of the direct evolution assay, were evaluated against *P. aeruginosa* Pa3GrPv cells to determine whether the evolved phages exhibited enhanced and/or prolonged bacterial growth suppression over a 24 h period compared to their wild-type counterparts.

**Figure 9:**
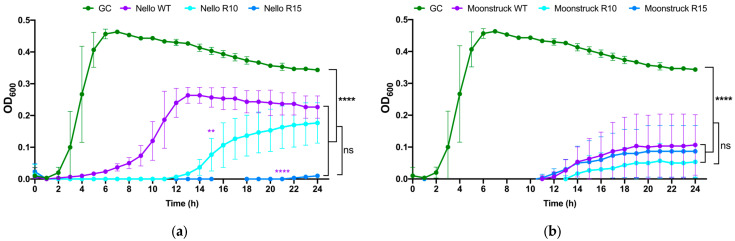
Kinetics of lysis curves of phage (**a**) Nello and (**b**) Moonstruck and their mutants obtained from round 10 (R10) and 15 (R15), with wild-type phages and growth controls (GC) as reference. On the y-axis is reported the optical density at 600 nm (OD_600_), the values representing the bacterial load. On the x-axis is represented the time-point in hours. One-way ANOVA multiple comparisons with Tukey correction were performed to compare all conditions with each other at each hour. ** *p* ≤ 0.01; **** *p* ≤ 0.0001; ns *p* > 0.05.

As shown in Figure 9a, the growth curves of *P. aeruginosa* Pa3GrPv treated with phage Nello, both wild-type and evolved variants from rounds 10 (R10) and 15 (R15), showed that the presence of wild-type Nello suppressed bacterial growth for up to 4 h, whereas Nello R10 extended this suppression to 12 h. Notably, Nello R15 maintained control of the bacterial load for 24 h. These results suggest that Nello R10 and Nello R15 strongly inhibited the bacterial growth resulting in a statistically significant difference to the wild-type Nello at 24 h (*p* ≤ 0.01 and *p* ≤ 0.0001, respectively). In addition, treatments with Nello wild-type, Nello R10 and R15 also showed statistically significant difference in the bacterial load at 24 h, compared to the growth control (*p* ≤ 0.0001).

However, no significant difference was observed between Nello R10 and R15 treatments, indicating similar lytic performance between these evolved variants. Similarly, as shown in Figure 9b, all treatments with phage Moonstruck (wild-type, R10, and R15) were able to suppress the bacterial growth up to 12 h and no statistically significant difference was observed among the Moonstruck variants at 24 h. For all the treatments with the phages, the OD_600_ measured at 24 h was different from that observed for the growth control (*p* ≤ 0.0001). The R10 and R15 variants exhibited comparable lytic curve profiles in respect of the wild-type, with no statistically significant difference in the duration of bacterial growth suppression.

### 3.6. Studying of the Mutations Insurged in the Evolved Phages

Given that the evolved phages Nello and Moonstruck from round 10 showed improved lytic activity against the clinical isolate Pa3GrPv compared to their wild-type counterparts, genome sequencing was performed to identify specific mutations within their coding sequences. Genomic DNA was extracted and sequenced for both phage lysates of round 10 and 15 of evolution. Since the evolved phage lysate from the first round of evolution likely consisted of a heterogeneous phage population, individual plaques were isolated and selected and were analyzed separately. For phages of round 15 of evolution, no plaque isolation was performed at the end of the fifteenth round of evolution to identify mutations fixed in the evolved phage population. Genomes were analyzed for mutations relative to the wild-type phage sequence. The mutations identified in phages isolated from round 10 of Nello and Moonstruck are listed in Table 3 and Table 4, respectively.

In the genomes of plaque 1, 4, and 5 of phage Nello, an SNP at position 29,406 (G>T) was found in a gene encoding for a protein annotated as DUF2612 domain-containing protein, resulting in a glycine-to-cysteine substitution at amino acid position 76. In the same plaques, two additional mutations were identified within a gene encoding a tail protein: an SNP at position 33,406 (A>T), and a complex mutation at position 33,554 involving the deletion of “AC” and insertion of “GA”. Plaque 1 also exhibited a unique SNP at position 33,416 (C>T), resulting in a proline-to-leucine substitution at amino acid position 906 of the tail protein. Meanwhile, plaques 4 and 5 showed another SNP at position 33,419 (C>T), which caused a threonine-to-isoleucine substitution at position 907 in the same gene. An SNP at position 49,018 (C>T), present in plaques 1 and 2, affected a gene encoding a putative polyamine oxidase, causing a glycine-to-aspartic acid substitution at amino acid position 56. A deletion at position 49,221 (TA>T) was found in an intergenic region in plaques 3, 4, and 5. Lastly, plaque 5 carried an SNP at position 59,798 (A>G) in a gene encoding a protein of unknown function.

Fewer mutations were identified in the Moonstruck evolved variants. Three mutations were detected within a single gene encoding a hypothetical protein with no predicted function: an insertion at position 73,289 (T>TGCG), a SNP at 73,297 (C>A), and another SNP at 73,054 (G>A). These mutations resulted in the following amino acid changes: glutamic acid to aspartic acid–alanine (position 60), glycine to tryptophan (position 58), and proline to serine (position 139), respectively. The first two were observed in plaque 1, and the last in plaque 5. In plaque 2, an SNP at position 21,885 (G>C) led to a glycine-to-arginine substitution at position 659 in a tail fiber protein. Plaque 4 harbored an insertion at position 69,936 (C>CG), resulting in a frameshift mutation in a gene encoding a hypothetical protein. No mutations were detected in plaque 3 of Moonstruck R10.

**Figure 10:**
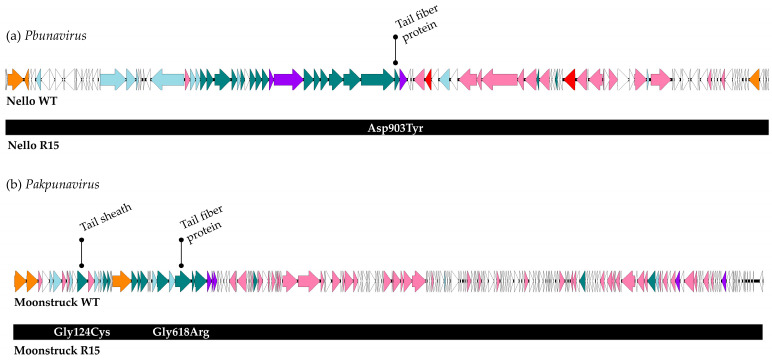
For each bacteriophage genus, a genome map is provided for the ancestral phage ((**a**) Nello and (**b**) Moonstruck) with the relevant functional annotations highlighted on top. Each colored arrow represents a coding sequence with white encoding hypothetical proteins, pink (DNA) metabolism-associated proteins, light-blue virion proteins, orange viral packaging, and purple cell lysis proteins. The genomic changes in the evolved phages after round 15 are displayed below each ancestral phage genome.

A schematic overview of the mutations identified at the end of the direct evolution process (round 15) is presented in Figure 10.

For phage Nello, the only mutation identified at the end of the evolution process was a single nucleotide polymorphism (SNP) at position 33,406, resulting in an amino acid substitution from asparagine to tyrosine at position 903 in a tail protein. In the genome of Moonstruck from round 15, two SNPs were detected. The first, at position 8182 (G>T), occurred within a gene encoding a tail sheath protein and resulted in a glycine-to-cysteine substitution at amino acid position 124. The second SNP, at position 21,762 (G>A), led to a glycine-to-arginine substitution at position 618 in a gene encoding a tail fiber protein. Notably, this gene was also found to be mutated in plaque 2 of Moonstruck R10.

## 4. Discussion

In recent years, phage therapy has become a widely recognized alternative for fighting pathogenic bacteria, including *Pseudomonas aeruginosa* [30,31]. The use of phages as an alternative or complement to antibiotics in the treatment of difficult-to-treat bacterial infections is a re-emerging strategy to fight antimicrobial resistance [32]. Therapeutic phages, isolated from the environment, could efficiently be employed for the treatment of bacterial infection either by combining them with antibiotics or enhancing their lytic activity adapting them against a specific bacterial strain [33,34]. In fact, previous research has already shown how bacteriophages could work synergistically in combination with antibiotics to eradicate bacterial infections [35] and how an initially weak killing activity of a phage against a clinical isolate of interest could be improved by directed evolution assays [33,36,37,38]. Here, we isolated and characterized three new phages (Cisa, Nello, and Moonstruck) from the environment. Genomic analysis revealed that all are myoviruses; in particular, phage Cisa and Nello are *Pbunaviruses*, while Moonstruck belongs to the *Pakpunavirus* genus. Moonstruck was classified as a new phage species, with a sequence similarity of 93.53% to known phages already present in public databases. None of the newly isolated phages carry undesirable genes like antibiotic resistance determinants, virulence factors or toxins, making them safe for therapy [7]. Moreover, in their genome no genes linked with lysogeny were identified, indicating a virulent nature. This was also observed for other *Pbunavirus* and *Pakpunavirus* environmental isolates [39,40,41,42]. Both these bacteriophage genera were previously described and have shown good in vitro therapeutic potential to treat *P. aeruginosa* infections [43,44,45,46]. Phenotypically, phages Nello and Moonstruck showed growth parameters comparable to similar already-described phages, with a latent period of 10 and 20 min, reaching a plateau around 10^7^ PFU/mL and 10^10^ PFU/mL, respectively [42,47].

Regarding thermal and pH stability, phages Nello and Moonstruck exhibited robust profiles, since their stability was not compromised when exposed to extreme temperatures and pH values. Phage Moonstruck exhibited a stable titer for temperature up to 60 °C, comparable to other *Pseudomonas* bacteriophages [31,48,49]. However, phage Nello showed a higher thermal stability (70 °C), similar to other myoviruses belonging to the *Pbunavirus* genus [49,50,51,52]. Moreover, both phages showed a high stability for extreme pH values, with phage Nello stable from pH 3 to 12 [48,53] and Moonstruck losing efficacy only at pH 3 [49,54]. Phage stability is a key factor to consider when selecting a phage for therapeutic application, because the phage preparation must be preserved for a long time without losing its infective capacity and because the administration of the phage preparation can take place in different body sites where the pH could vary [7]. In order to treat a patient candidate for phage therapy with a lung infection caused by *P*. *aeruginosa* Pa3GrPv, phages Nello and Moonstruck were tested alone and in combination with ciprofloxacin to assess potential synergistic and additive interactions. As previously described in the literature, phage–antibiotic combinations may enhance the lytic potential of phages, even possibly preventing the selection of resistant bacteria, achieving eradication [34,55]. In our study, the combination of Moonstruck and the ¼ MIC value of ciprofloxacin (0.125 µg/mL) showed no bacterial growth up to 48 h while the phage alone was able to control the bacterial load up to 12 h. For phage Nello, we observed a slight difference compared to Moonstruck. In particular, the combination of Nello with ciprofloxacin (0.125 µg/mL) was better in controlling the bacterial growth of either the phage or antibiotic alone, suggesting an additive effect, even showing increasing optical density values overtime.

Another method to improve phage lytic efficacy is the directed evolution approach, consisting in the adaptation of the phage to the target bacterial strain co-evolving them and then isolating an evolved phage variants to use against the wild-type bacteria [10]. In this work a 5-day evolution protocol was employed since, as observed by Tkhilaishvili et al., a longer period of incubation improves lytic activity against bacteria [27].

This observation may be associated with the selection of phage mutants with a better killing activity as prolonged exposure, characterized by a rise in phage number during bacterial lysis, might facilitate the interaction of phages with bacterial cells.

The batch culture-based selection method is the most used for phage training: bacteria and phages are co-cultured either in liquid or on agar plates for a certain time and successively the mutant phages are isolated and characterized. The time of co-evolution varies extensively according to the model used: the selection of mutants can be performed after a “one-time” interaction between phages and bacteria that can be relatively brief (overnight or 24 h), or leaving the phage and bacteria to continuously interact for an extended number of days [56]. In this study, a directed evolution protocol consisting of 5 days of co-incubation was independently performed on phage Nello and Moonstruck against the clinical isolate Pa3GrPv. Both the 24 h phage treatment and the lysis kinetics revealed an improved lytic ability of the evolved variants of phage Nello showing a progressively improved lytic ability against the isolate, compared to the wild-type phage. Conversely, the trained clones of phage Moonstruck did not show an enhanced infection ability for the Pa3GrPv strain compared to the ancestral phage. The behavior of the evolved phage Nello is aligned with what has been previously described in other cases of phage-adaptation, both for Gram-negative [10,33,57] and Gram-positive bacteria [37,58,59], where the adapted phages exhibited a higher ability to control the bacterial load. This evidence suggests how bacteria are less prone to evolve resistance against trained phages. However, evolutionary dynamic outcomes could show several variations and are not easily predictable, even in the simplest setup of a single strain challenged with a unique phage isolate [60]. Contrary to what was observed for Nello, Moonstruck did not show a consistent improvement throughout the adaptation process and this finding was also reported by Betts et al., who described how certain trained phages were less infectious to co-evolved bacteria [60]. This phenomenon could be linked with continuous co-evolution, in which a fraction of the bacterial population retains phage sensitivity. On one hand, it can arise since phages continually evolve, becoming active against resistant bacteria; on the other hand, it may rely on a heterogeneous bacterial population of both resistant and sensitive bacterial clones, as a result of the high cost associated with resistance [61]. This concept could also be described by a co-evolutionary arms race (ARD) and fluctuating selection (FSD), terms used to describe host–parasite co-evolution. Under ARD, directional selection predominates and leads to the accumulation of infectivity and resistance genes, allowing parasites to infect all past host genomes. In FSD, there are temporal fluctuations in infectivity and resistance genes rather than an accumulation, resulting in unchanged infectivity of the parasite [60]. Persistent co-evolution has been reported for phage–bacteria systems where the bacterial culture was maintained throughout the experiment. Instead, an unstable coexistence was observed when evolved phages were used to treat a fresh bacterial culture at every transfer [61]. This could suggest that, for Moonstruck, the applied protocol could involve a too long incubation of phage and bacteria for each round. Moreover, on average, Moonstruck had a 2–3 log_10_ increment from the initial phage titer in each round, while Nello showed a higher increase of 3–4 log_10_ PFU/mL. This could suggest that Moonstruck requires more evolution rounds to accumulate and select mutations. Esvelt et al. reported that a direct evolution allows the generation of mutations using distinct evolutionary trajectories, which might converge and end up in proteins sharing the same function [62]. In particular, following a direct evolution protocol, point mutations could emerge in genes encoding for important proteins involved in host recognition and/or attachment [30]. So, to understand the molecular basis of the results obtained from the study of the lytic activity of phages of round ten 10 and 15, and identify possible key genes involved in phage–bacteria interaction, genome sequencing and mutations analysis were performed. The results showed that some viral clones had developed spontaneous mutations which could have potentially led to the improvement in their lytic activity. For both phages, the genes showing point mutations encoding for tail proteins. This kind of mutation was already reported in other articles which studied the genomic modification insurged at the end of the phage adaptation [33,37,59]. Regarding phage Nello, the mutated tail protein showed a high similarity (100% query and 98.03% coverage) with the tail protein of *Pseudomonas* phage E217 (gp46), the structure of which has been extensively characterized using Cryo-EM [63]. This tail protein assembles into a trimer that attaches with the N-terminal 340 amino acids to the baseplate. In contrast, the flexible C-terminal region makes contact with the host O-antigen via a lectin-binding domain, resulting in phage attachment to the bacteria surface [63]. The observed substitution of a negatively charged amino acid, such as aspartic acid, with a nonpolar residue like tyrosine in the tail protein of the evolved phage may have enhanced the interaction between the protein and the anionic lipopolysaccharide [64,65]. The study of mutations obtained after phage–host interaction provides key information related to the infection mechanism of that specific phage against a specific bacterium and allows speculation as to which proteins are involved in the recognition and attachment to that host.

To conclude, this study highlights the growing potential of phage therapy as a viable strategy to combat antibiotic-resistant bacterial infections, particularly those caused by *P*. *aeruginosa*. The isolation and comprehensive characterization of three new environmental phages, including the novel species Moonstruck, expand the existing phage library with candidates for therapeutic application. Our results confirm that these phages, free from undesirable genes, can exert a significant antibacterial effect, especially when used in combination with antibiotics such as ciprofloxacin.

Furthermore, through a directed evolution protocol, we were able to enhance the lytic activity of phage Nello against the target clinical isolate. This reinforces the contribution of phage training as a tool for tailoring phage infectivity and overcoming initial limitations in lytic efficacy. Conversely, the lack of consistent improvement observed in phage Moonstruck highlights the complexity and unpredictability of co-evolutionary dynamics, suggesting that optimization strategies may need to be phage-specific. Moreover, it is fundamental to recognize that evolution protocols are time-consuming and, therefore, they may be more appropriate to target strains causing chronic infections rather than acute cases. Based on our laboratory experience, identifying an active phage from a collection of pre-characterized bacteriophages typically requires about one week, while the isolation of a new phage may take two to four weeks. Meanwhile, the adaptation protocol described in this study required approximately ten weeks to complete. These timelines underscore the need to carefully evaluate the clinical context when considering the application of phage therapy, particularly for the urgency of treatment.

Overall, our findings contribute to the comprehension of phage–host interactions, the role of evolutionary adaptation in improving phage efficacy, and the importance of environmental reservoirs in the discovery of new therapeutic phages.

## Figures and Tables

**Table 1:** Genomic features of the three bacteriophages isolated from water samples.

Phage Name	Length (bp)	GC Content (%)	Genus	BLAST Best Hit	Query Coverage	Identity
Cisa	65,883	54.95	*Pbunavirus*	misfit	100%	97.18%
Nello	65,886	54.96	*Pbunavirus*	misfit	100%	97.18%
Moonstruck	92,882	49.29	*Pakpunavirus*	PAK_P4	97%	96.42%

**Table 2:** Summary of the characteristics of the genomes after annotation.

Phage Name	Accession Number	CDS	tRNAs	Proteins with Predicted Functions	Hypothetical Proteins
Cisa	PV464114	107	-	46	56
Nello	PV464115	107	-	50	57
Moonstruck	PV464116	199	12	77	122

**Table 3:** List of all the non-silent mutations identified in Nello mutants from Round 10.

Mutant	Position (bp)	Mutation Type	nt Mutation	AA Substitution	Gene Product	Protein Function
P1	29,406	SNP	G>T	Gly76Cys	gp 48	DUF2612 domain-containing protein
	33,406	SNP	A>T	Asn903Tyr	gp 49	Tail protein
	33,416	SNP	C>T	Pro906Leu	gp 49	Tail protein
	33,554	Complex	AC>GA	Asn952Arg	gp 49	Tail protein
	49,018	SNP	C>T	Gly56Asp	gp 71	Putative polyamine oxidase
P2	49,018	SNP	C>T	Gly56Asp	gp 71	Putative polyamine oxidase
P3	49,221	Deletion	TA>T	-	-	Intergenic region
P4	29,406	SNP	G>T	Gly76Cys	gp 48	DUF2612 domain-containing protein
	33,406	SNP	A>T	Asn903Tyr	gp 49	Tail protein
	33,419	SNP	C>T	Thr907Ile	gp 49	Tail protein
	33,554	Complex	AC>GA	Asn952Arg	gp 49	Tail protein
	49,221	Deletion	TA>T	-	-	Intergenic region
P5	29,406	SNP	G>T	Gly76Cys	gp 48	DUF2612 domain-containing protein
	33,406	SNP	A>T	Asn903Tyr	gp 49	Tail protein
	33,419	SNP	C>T	Thr907Ile	gp 49	Tail protein
	33,554	Complex	AC>GA	Asn952Arg	gp 49	Tail protein
	49,221	Deletion	TA>T	-	-	Intergenic region
	59,798	SNP	A>G	Lys45Lys	gp 89	Hypothetical protein

**Table 4:** List of all the non-silent substitutions identified in Moonstruck mutants from Round 10.

Mutant	Position (bp)	Mutation Type	nt Mutation	AA Substitution	Gene Product	Protein Function
P1	73,289	Insertion	T>TGCG	Glu60del insAspAla	gp154	Hypothetical protein
	73,297	SNP	C>A	Gly58Trp	gp154	Hypothetical protein
P2	21,885	SNP	G>C	Gly659Arg	gp31	Tail fiber protein
P4	69,936	Insertion	C>CG	Glu18fs	gp145	Hypothetical protein
P5	73,054	SNP	G>A	Pro139Ser	gp154	Hypothetical protein

## Data Availability

The assembled and annotated genomes were submitted to the GenBank, and the following accession numbers were used: PV464114 for phage Cisa, PV464115 for phage Nello and PV464116 for phage Moonstruck.

## Supplementary Materials

The following supporting information can be downloaded at: https://www.mdpi.com/article/10.3390/v17070938/s1, Table S1: List of genomic differences between the two *Pbunavirus* phages Cisa and Nello.

## References

[1] Strathdee S.A., Hatfull G.F., Mutalik V.K., Schooley R.T. (2023). Phage Therapy: From Biological Mechanisms to Future Directions. Cell.

[2] Ruekit S., Srijan A., Serichantalergs O., Margulieux K.R., Mc Gann P., Mills E.G., Stribling W.C., Pimsawat T., Kormanee R., Nakornchai S. (2022). Molecular Characterization of Multidrug-Resistant ESKAPEE Pathogens from Clinical Samples in Chonburi, Thailand (2017–2018). BMC Infect. Dis..

[3] Gellatly S.L., Hancock R.E.W. (2013). *Pseudomonas aeruginosa*: New Insights into Pathogenesis and Host Defenses. Pathog. Dis..

[4] Pang Z., Raudonis R., Glick B.R., Lin T.-J., Cheng Z. (2019). Antibiotic Resistance in *Pseudomonas aeruginosa*: Mechanisms and Alternative Therapeutic Strategies. Biotechnol. Adv..

[5] Munita J.M., Arias C.A. (2016). Mechanisms of Antibiotic Resistance. Microbiol. Spectr..

[6] Luong T., Salabarria A.-C., Roach D.R. (2020). Phage Therapy in the Resistance Era: Where Do We Stand and Where Are We Going?. Clin. Ther..

[7] Hatfull G.F., Dedrick R.M., Schooley R.T. (2022). Phage Therapy for Antibiotic-Resistant Bacterial Infections. Annu. Rev. Med..

[8] Friman V., Soanes-Brown D., Sierocinski P., Molin S., Johansen H.K., Merabishvili M., Pirnay J., De Vos D., Buckling A. (2016). Pre-adapting Parasitic Phages to a Pathogen Leads to Increased Pathogen Clearance and Lowered Resistance Evolution with *Pseudomonas aeruginosa* Cystic Fibrosis Bacterial Isolates. J. Evol. Biol..

[9] Abdelsattar A., Dawooud A., Rezk N., Makky S., Safwat A., Richards P., El-Shibiny A. (2021). How to Train Your Phage: The Recent Efforts in Phage Training. Biologics.

[10] Borin J.M., Avrani S., Barrick J.E., Petrie K.L., Meyer J.R. (2021). Coevolutionary Phage Training Leads to Greater Bacterial Suppression and Delays the Evolution of Phage Resistance. Proc. Natl. Acad. Sci. USA.

[11] Azeredo J., Sillankorva S., Pires D.P. (2014). *Pseudomonas* Bacteriophage Isolation and Production. Methods Mol. Biol..

[12] Kropinski A.M., Mazzocco A., Waddell T.E., Lingohr E., Johnson R.P., Clokie M.R.J., Kropinski A.M. (2009). Enumeration of Bacteriophages by Double Agar Overlay Plaque Assay. Bacteriophages.

[13] Turchi B., Campobasso C., Nardinocchi A., Wagemans J., Torracca B., Lood C., Di Giuseppe G., Nieri P., Bertelloni F., Turini L. (2024). Isolation and Characterization of Novel *Staphylococcus aureus* Bacteriophage Hesat from Dairy Origin. Appl. Microbiol. Biotechnol..

[14] Wick R.R., Judd L.M., Gorrie C.L., Holt K.E. (2017). Unicycler: Resolving Bacterial Genome Assemblies from Short and Long Sequencing Reads. PLoS Comput. Biol..

[15] Sullivan M.J., Petty N.K., Beatson S.A. (2011). Easyfig: A Genome Comparison Visualizer. Bioinformatics.

[16] Golosova O., Henderson R., Vaskin Y., Gabrielian A., Grekhov G., Nagarajan V., Oler A.J., Quiñones M., Hurt D., Fursov M. (2014). Unipro UGENE NGS Pipelines and Components for Variant Calling, RNA-Seq and ChIP-Seq Data Analyses. PeerJ.

[17] Brettin T., Davis J.J., Disz T., Edwards R.A., Gerdes S., Olsen G.J., Olson R., Overbeek R., Parrello B., Pusch G.D. (2015). RASTtk: A Modular and Extensible Implementation of the RAST Algorithm for Building Custom Annotation Pipelines and Annotating Batches of Genomes. Sci. Rep..

[18] Merrill B.D., Ward A.T., Grose J.H., Hope S. (2016). Software-Based Analysis of Bacteriophage Genomes, Physical Ends, and Packaging Strategies. BMC Genom..

[19] Bortolaia V., Kaas R.S., Ruppe E., Roberts M.C., Schwarz S., Cattoir V., Philippon A., Allesoe R.L., Rebelo A.R., Florensa A.F. (2020). ResFinder 4.0 for Predictions of Phenotypes from Genotypes. J. Antimicrob. Chemother..

[20] Malberg Tetzschner A.M., Johnson J.R., Johnston B.D., Lund O., Scheutz F. (2020). In Silico Genotyping of *Escherichia coli* Isolates for Extraintestinal Virulence Genes by Use of Whole-Genome Sequencing Data. J. Clin. Microbiol..

[21] Joensen K.G., Scheutz F., Lund O., Hasman H., Kaas R.S., Nielsen E.M., Aarestrup F.M. (2014). Real-Time Whole-Genome Sequencing for Routine Typing, Surveillance, and Outbreak Detection of Verotoxigenic *Escherichia coli*. J. Clin. Microbiol..

[22] Alcock B.P., Huynh W., Chalil R., Smith K.W., Raphenya A.R., Wlodarski M.A., Edalatmand A., Petkau A., Syed S.A., Tsang K.K. (2023). CARD 2023: Expanded Curation, Support for Machine Learning, and Resistome Prediction at the Comprehensive Antibiotic Resistance Database. Nucleic Acids Res..

[23] Feldgarden M., Brover V., Gonzalez-Escalona N., Frye J.G., Haendiges J., Haft D.H., Hoffmann M., Pettengill J.B., Prasad A.B., Tillman G.E. (2021). AMRFinderPlus and the Reference Gene Catalog Facilitate Examination of the Genomic Links among Antimicrobial Resistance, Stress Response, and Virulence. Sci. Rep..

[24] Pires D.P., Monteiro R., Mil-Homens D., Fialho A., Lu T.K., Azeredo J. (2021). Designing *P. aeruginosa* Synthetic Phages with Reduced Genomes. Sci. Rep..

[25] Fujiki J., Nakamura T., Nakamura K., Nishida K., Amano Y., Watanabe Y., Gondaira S., Usui M., Shimizu M., Miyanaga K. (2022). Biological Properties of *Staphylococcus* Virus ΦSA012 for Phage Therapy. Sci. Rep..

[26] The European Committee on Antimicrobial Susceptibility Testing—EUCAST (2025). MIC Determination of Non-Fastidious and Fastidious Organisms.

[27] Tkhilaishvili T., Wang L., Tavanti A., Trampuz A., Di Luca M. (2020). Antibacterial Efficacy of Two Commercially Available Bacteriophage Formulations, Staphylococcal Bacteriophage and PYO Bacteriophage, Against Methicillin-Resistant *Staphylococcus aureus*: Prevention and Eradication of Biofilm Formation and Control of a Systemic Infection of *Galleria mellonella* Larvae. Front. Microbiol..

[28] Dion M.B., Oechslin F., Moineau S. (2020). Phage Diversity, Genomics and Phylogeny. Nat. Rev. Microbiol..

[29] Turner D., Kropinski A.M., Adriaenssens E.M. (2021). A Roadmap for Genome-Based Phage Taxonomy. Viruses.

[30] Laanto E., Mäkelä K., Hoikkala V., Ravantti J.J., Sundberg L.-R. (2020). Adapting a Phage to Combat Phage Resistance. Antibiotics.

[31] Sharma S., Datta S., Chatterjee S., Dutta M., Samanta J., Vairale M.G., Gupta R., Veer V., Dwivedi S.K. (2021). Isolation and Characterization of a Lytic Bacteriophage against *Pseudomonas aeruginosa*. Sci. Rep..

[32] Pirnay J.-P., Djebara S., Steurs G., Griselain J., Cochez C., De Soir S., Glonti T., Spiessens A., Vanden Berghe E., Green S. (2024). Personalized Bacteriophage Therapy Outcomes for 100 Consecutive Cases: A Multicentre, Multinational, Retrospective Observational Study. Nat. Microbiol..

[33] Kunisch F., Campobasso C., Wagemans J., Yildirim S., Chan B.K., Schaudinn C., Lavigne R., Turner P.E., Raschke M.J., Trampuz A. (2024). Targeting *Pseudomonas aeruginosa* Biofilm with an Evolutionary Trained Bacteriophage Cocktail Exploiting Phage Resistance Trade-Offs. Nat. Commun..

[34] Ferran A.A., Lacroix M.Z., Gourbeyre O., Huesca A., Gaborieau B., Debarbieux L., Bousquet-Mélou A. (2022). The Selection of Antibiotic- and Bacteriophage-Resistant *Pseudomonas aeruginosa* Is Prevented by Their Combination. Microbiol. Spectr..

[35] Duplessis C., Warawa J.M., Lawrenz M.B., Henry M., Biswas B. (2021). Successful Intratracheal Treatment of Phage and Antibiotic Combination Therapy of a Multi-Drug Resistant *Pseudomonas aeruginosa* Murine Model. Antibiotics.

[36] Eskenazi A., Lood C., Wubbolts J., Hites M., Balarjishvili N., Leshkasheli L., Askilashvili L., Kvachadze L., Van Noort V., Wagemans J. (2022). Combination of Pre-Adapted Bacteriophage Therapy and Antibiotics for Treatment of Fracture-Related Infection Due to Pandrug-Resistant *Klebsiella pneumoniae*. Nat. Commun..

[37] Ponce Benavente L., Wagemans J., Hinkel D., Aguerri Lajusticia A., Lavigne R., Trampuz A., Gonzalez Moreno M. (2024). Targeted Enhancement of Bacteriophage Activity against Antibiotic-Resistant *Staphylococcus aureus* Biofilms through an Evolutionary Assay. Front. Microbiol..

[38] Morello E., Saussereau E., Maura D., Huerre M., Touqui L., Debarbieux L. (2011). Pulmonary Bacteriophage Therapy on *Pseudomonas aeruginosa* Cystic Fibrosis Strains: First Steps Towards Treatment and Prevention. PLoS ONE.

[39] Campbell R.A., Farlow J., Freyberger H.R., He Y., Ward A.M., Ellison D.W., Getnet D., Swierczewski B.E., Nikolich M.P., Filippov A.A. (2021). Genome Sequences of 17 Diverse *Pseudomonas aeruginosa* Phages. Microbiol. Resour. Announc..

[40] Farlow J., Freyberger H.R., He Y., Ward A.M., Rutvisuttinunt W., Li T., Campbell R., Jacobs A.C., Nikolich M.P., Filippov A.A. (2020). Complete Genome Sequences of 10 Phages Lytic against Multidrug-Resistant *Pseudomonas aeruginosa*. Microbiol. Resour. Announc..

[41] Debarbieux L., Leduc D., Maura D., Morello E., Criscuolo A., Grossi O., Balloy V., Touqui L. (2010). Bacteriophages Can Treat and Prevent *Pseudomonas aeruginosa* Lung Infections. J. Infect. Dis..

[42] Akremi I., Merabishvili M., Jlidi M., Haj Brahim A., Ben Ali M., Karoui A., Lavigne R., Wagemans J., Pirnay J.-P., Ben Ali M. (2022). Isolation and Characterization of Lytic *Pseudomonas aeruginosa* Bacteriophages Isolated from Sewage Samples from Tunisia. Viruses.

[43] Henry M., Lavigne R., Debarbieux L. (2013). Predicting In Vivo Efficacy of Therapeutic Bacteriophages Used To Treat Pulmonary Infections. Antimicrob. Agents Chemother..

[44] Fukuda K., Ishida W., Uchiyama J., Rashel M., Kato S., Morita T., Muraoka A., Sumi T., Matsuzaki S., Daibata M. (2012). *Pseudomonas aeruginosa* Keratitis in Mice: Effects of Topical Bacteriophage KPP12 Administration. PLoS ONE.

[45] Parra B., Sandoval M., Arriagada V., Amsteins L., Aguayo C., Opazo-Capurro A., Dechesne A., González-Rocha G. (2024). Isolation and Characterization of Lytic Bacteriophages Capable of Infecting Diverse Multidrug-Resistant Strains of *Pseudomonas aeruginosa*: PaCCP1 and PaCCP2. Pharmaceuticals.

[46] Forti F., Roach D.R., Cafora M., Pasini M.E., Horner D.S., Fiscarelli E.V., Rossitto M., Cariani L., Briani F., Debarbieux L. (2018). Design of a Broad-Range Bacteriophage Cocktail That Reduces *Pseudomonas aeruginosa* Biofilms and Treats Acute Infections in Two Animal Models. Antimicrob. Agents Chemother..

[47] Aghaee B.L., Khan Mirzaei M., Alikhani M.Y., Mojtahedi A., Maurice C.F. (2021). Improving the Inhibitory Effect of Phages against *Pseudomonas aeruginosa* Isolated from a Burn Patient Using a Combination of Phages and Antibiotics. Viruses.

[48] Guo Y., Chen P., Lin Z., Wang T. (2019). Characterization of Two *Pseudomonas aeruginosa* Viruses vB_PaeM_SCUT-S1 and vB_PaeM_SCUT-S2. Viruses.

[49] Nour El-Din H.T., Kettal M., Granados Maciel J.C., Beaudoin G., Oktay U., Hrapovic S., Sad S., Dennis J.J., Peters D.L., Chen W. (2025). Isolation, Characterization, and Genomic Analysis of Bacteriophages Against *Pseudomonas aeruginosa* Clinical Isolates from Early and Chronic Cystic Fibrosis Patients for Potential Phage Therapy. Microorganisms.

[50] Oliveira V.C., Bim F.L., Monteiro R.M., Macedo A.P., Santos E.S., Silva-Lovato C.H., Paranhos H.F.O., Melo L.D.R., Santos S.B., Watanabe E. (2020). Identification and Characterization of New Bacteriophages to Control Multidrug-Resistant *Pseudomonas aeruginosa* Biofilm on Endotracheal Tubes. Front. Microbiol..

[51] Wannasrichan W., Htoo H.H., Suwansaeng R., Pogliano J., Nonejuie P., Chaikeeratisak V. (2022). Phage-Resistant *Pseudomonas aeruginosa* against a Novel Lytic Phage JJ01 Exhibits Hypersensitivity to Colistin and Reduces Biofilm Production. Front. Microbiol..

[52] Camens S., Liu S., Hon K., Bouras G.S., Psaltis A.J., Wormald P.-J., Vreugde S. (2021). Preclinical Development of a Bacteriophage Cocktail for Treating Multidrug Resistant *Pseudomonas aeruginosa* Infections. Microorganisms.

[53] Xuan G., Kong J., Wang Y., Lin H., Wang J. (2023). Characterization of the Newly Isolated *Pseudomonas* Phage vB_Pae_LC3I3. Virus Res..

[54] Kong J., Xuan G., Lin H., Wang J. (2023). Characterization of a Novel Phage vB_Pae_HB2107-3I That Infects *Pseudomonas aeruginosa*. Mol. Genet. Genom..

[55] Cesta N., Pini M., Mulas T., Materazzi A., Ippolito E., Wagemans J., Kutateladze M., Fontana C., Sarmati L., Tavanti A. (2023). Application of Phage Therapy in a Case of a Chronic Hip-Prosthetic Joint Infection Due to *Pseudomonas aeruginosa*: An Italian Real-Life Experience and In Vitro Analysis. Open Forum Infect. Dis..

[56] Oyejobi G.K., Zhang X., Xiong D., Ogolla F., Xue H., Wei H. (2023). Phage-Bacterial Evolutionary Interactions: Experimental Models and Complications. Crit. Rev. Microbiol..

[57] Akusobi C., Chan B.K., Williams E.S.C.P., Wertz J.E., Turner P.E. (2018). Parallel Evolution of Host-Attachment Proteins in Phage PP01 Populations Adapting to *Escherichia coli* O157:H7. Pharmaceuticals.

[58] Sergueev K.V., Filippov A.A., Farlow J., Su W., Kvachadze L., Balarjishvili N., Kutateladze M., Nikolich M.P. (2019). Correlation of Host Range Expansion of Therapeutic Bacteriophage Sb-1 with Allele State at a Hypervariable Repeat Locus. Appl. Environ. Microbiol..

[59] Sáez Moreno D., Visram Z., Mutti M., Restrepo-Córdoba M., Hartmann S., Kremers A.I., Tišáková L., Schertler S., Wittmann J., Kalali B. (2021). Ε2-Phages Are Naturally Bred and Have a Vastly Improved Host Range in *Staphylococcus aureus* over Wild Type Phages. Pharmaceuticals.

[60] Betts A., Kaltz O., Hochberg M.E. (2014). Contrasted Coevolutionary Dynamics between a Bacterial Pathogen and Its Bacteriophages. Proc. Natl. Acad. Sci. USA.

[61] Brockhurst M.A., Buckling A., Rainey P.B. (2006). Spatial Heterogeneity and the Stability of Host-Parasite Coexistence. J. Evol. Biol..

[62] Esvelt K.M., Carlson J.C., Liu D.R. (2011). A System for the Continuous Directed Evolution of Biomolecules. Nature.

[63] Li F., Hou C.-F.D., Lokareddy R.K., Yang R., Forti F., Briani F., Cingolani G. (2023). High-Resolution Cryo-EM Structure of the *Pseudomonas* Bacteriophage E217. Nat. Commun..

[64] Biro J.C. (2006). Amino Acid Size, Charge, Hydropathy Indices and Matrices for Protein Structure Analysis. Theor. Biol. Med. Model..

[65] Zhang L., Dhillon P., Yan H., Farmer S., Hancock R.E.W. (2000). Interactions of Bacterial Cationic Peptide Antibiotics with Outer and Cytoplasmic Membranes of *Pseudomonas aeruginosa*. Antimicrob. Agents Chemother..

