# Supplementary material for: Therapeutic Optimization of Pseudomonas aeruginosa Phages: From Isolation to Directed Evolution
Source: Viruses. 2025 Jun 30;17(7):938. doi: 10.3390/v17070938 (PMC12300175; doi:10.3390/v17070938)
Supplement: Supplementary file 1 [file viruses-17-00938-s001.zip › viruses-3682309-supplementary.pdf]

Table S1. List of genomic differences between the two *Pbunavirus* phages Cisa and Nello

Position	Mutation	Cisa	Nello	Effect	Product
797	SNP	G	T	Missense variant 598 G>T Ala200Ser	Phage terminase
2521	INS	G	GC	Frameshift variant 45_46insG Pro16fs	Hypothetical protein
6994	SNP	A	C	Missense variant 6 T>G Asn2Lys	Hypothetical protein
7351	SNP	T	C	Missense variant 178 A>G Thr60Ala	Hypothetical protein
7512	SNP	A	G	Missense variant 17 C>T Val6Ala	Hypothetical protein
14565	SNP	C	A	Missense variant 835 G>T Ala279Ser	Hypothetical protein
19817	SNP	G	A	Missense variant 292 G>A Val98Ile	Phage tail fiber
20620	SNP	C	T	Missense variant 323 C>T Thr108Ile	Phage baseplate wedge subunit
23644	SNP	A	G	Missense variant 472 A>G Ile158Val	Phage lytic tail
45026	SNP	C	A	Missense variant 821 G>T Ser274Ile	3′-phosphatase, 5′-polynucleotide kinase
48457	SNP	T	C	Missense variant 728 A>G Asp243Gly	Hypothetical protein
57683	DEL	AT	A	Missense variant 35delT Leu12fs	Hypothetical protein
59833	SNP	T	C	Missense variant 98 A>G Gln33Arg	Hypothetical protein
60574	SNP	A	G	Missense variant 11 T>C Ile4Thr	Phage structural protein
61572	SNP	G	A	Missense variant 125 C>T Ser42Phe	Hypothetical protein
65336	SNP	T	G	Missense variant 81 A>C Leu27Phe	Hypothetical protein
65828	INS	C	CT	Frameshift variant stop_gained 55_56ins A Trp19fs	Hypothetical protein
